# Biologic Therapies Decrease Disease Severity and Improve Depression and Anxiety Symptoms in Psoriasis Patients

**DOI:** 10.3390/life13051219

**Published:** 2023-05-19

**Authors:** Teodora-Larisa Timis, Lehel Beni, Teodora Mocan, Ioan-Alexandru Florian, Remus-Ioan Orasan

**Affiliations:** 1 Department of Physiology, “Iuliu Hatieganu” University of Medicine and Pharmacy, 400012 Cluj-Napoca, Romania; 2 Department of Neurosciences, “Iuliu Hatieganu” University of Medicine and Pharmacy, 400012 Cluj-Napoca, Romania

**Keywords:** psoriasis, depression, anxiety, biologic agents, PASI, DLQI, severity

## Abstract

Background: Psoriasis is an immune-mediated chronic skin disease that is associated with a significant psychological burden. A newer line of therapy is represented by biologic agents. Our study aimed to evaluate the effect of biologic therapies in the treatment of psoriasis concerning both disease severity and psychological comorbidity. Material and Methods: We performed a prospective case-control comparison to evaluate the prevalence of depression and anxiety in psoriasis patients and unaffected individuals. All patients were recruited between October 2017 and February 2021. Baseline depression (PHQ-9), anxiety (GAD-7), PASI, and DLQI scores were noted. Then, we evaluated the efficacy of biologic treatment in reducing these scores at 6 months of therapy. Patients were treated with either ixekizumab, secukinumab, guselkumab, certolizumab, ustekinumab, risankizumab, or adalimumab. Results: 106 bio-naïve patients with psoriasis and 106 controls without the disease were included in this study. Depression and anxiety were significantly more common among psoriasis patients than in unaffected individuals (*p* < 0.0001). Female patients presented both depression and anxiety more frequently than men in both case and control groups. Disease severity was significantly associated with worsened depression and anxiety symptoms. Biologic therapy resulted in a significant decrease in all four scores at the 6-month mark for each patient (*p* < 0.0001). Only an improved PASI correlated significantly with lower depression and anxiety scores (*p* < 0.005), whereas a decreased DLQI did not (*p* > 0.955). None of the seven biologic agents used was discovered to be superior. Conclusion: biologic therapies are effective in decreasing both disease severity and alleviating depression and anxiety symptoms in psoriasis.

## 1. Introduction

Psoriasis represents a chronic, immune-mediated disease that is characterized by burdensome psychological comorbidities markedly exceeding typical clinical manifestations [1], including affected skin that has hypertrophied, is scaly in appearance, and is generally intensely pruriginous. Typical cutaneous lesions may arise on any area of the skin, usually on the extensor surfaces of the limbs, the scalp, or the lumbosacral area, yet those that are readily visible can elicit frustration and a negative self-image, affecting the quality of life and eventually surmounting to depression, anxiety, and even suicidal ideation [2]. Moreover, joint involvement is associated with an increased depressive burden [3]. Through a dysfunction in the hypothalamic–pituitary–adrenal (HPA) axis and an increased release of pro-inflammatory cytokines, stress plays a crucial role in both the onset and exacerbation of psoriasis [4]. Cutaneous inflammatory response subsequently generates anxiety/depression feelings through physical deformity and stigmatization. Furthermore, the irrational fear and apprehension that the disease may be contagious can lead to stigmatization and isolation of patients during school, work, or daily activities [5]. This traps psoriasis sufferers in a vicious cycle, with stress and depression provoking or intensifying psoriasis flares, which may in turn generate or exacerbate further stress and despair.

Proinflammatory cytokines have been implicated in the development and pathophysiology of anxiety and depression. It has been demonstrated that in psoriasis a immunologic cascade is activated, resulting in the overexpression of several proinflammatory cytokines that sustain and worsen the inflammatory process and psoriatic lesions. Among these, we recount tumor necrosis factor-alpha (TNF-a) and interleukins (IL)-6, IL-12, IL-23, and IL-17. It has also been observed that TNF-a activates the hypothalamic-pituitary-adrenal (HPA) axis, which leads to the depletion of tryptophane and the subsequent decrease in serotonin (5-HT) levels and the onset of depression [6]. Additionally, increased levels of TNF-a, IL-6, IL-17, and IL-23 were discovered in the serum of depressive patients [7]. The most recent advancement in the treatment of psoriasis is represented by proteins targeting specific components of the pathophysiological cascade [8,9,10,11]. These are mainly divided mainly based on their molecular targets, such as TNF-a inhibitors, IL-23, and IL-17 antagonists.

The first main objective of the following study was to compare the psychological characteristics of psoriasis patients and an equal number of controls. The second principal goal was to prospectively determine the efficacy of biologic agents in reducing both the severity of psoriasis and the psychological burden as assessed by specific scales for depression and anxiety. As a secondary objective, we aimed to test if any of the biologic agents used proved to be superior. 

## 2. Materials and Methods

### 2.1. Patients and Controls

We enrolled 106 patients with psoriasis and 106 disease-free controls to evaluate the differences in depression and anxiety between individuals affected by psoriasis and the general population. For every psoriasis patient, we attempted to select a suitable control of matching gender, background, and age category. All psoriasis patients were bio-naïve and under no psychological therapy either before or during biological treatment. Cases and controls were selected so as not to have other significant or severe pathologies that could impact their psychological profile. Psoriasis Area and Severity Index (PASI) and Dermatology Life Quality Index (DLQI) scores were calculated by the treating physician. The patients were prospectively evaluated both at the start of biologic therapy and at the 6-month treatment mark. All patients and controls were evaluated by the same two dermatologists (T.L.T. and R.I.O.) within a single private practice facility.

Depression was evaluated with the use of the Patient Health Questionnaire (PHQ-9), which assigns a severity index according to the score obtained on each question [12]. Likewise, the Generalized Anxiety Disorder 7-item (GAD-7) questionnaire was used to test symptoms of anxiety [13]. The initial questionnaires were administered by the main author starting from October 2017 to February 2021. These were then verified with each individual patient and control. Further, the severity scores were converted to ordinate numerical values: from 0 to 5 for depression, and from 0 to 3 for anxiety (Table 1).

Additionally, for psoriatic patients, we tested the age and duration of the disease for impact on depression and anxiety. PASI and DLQI were also compared before and at the 6-month treatment mark for biologic therapies. Those biologic therapies utilized and their assigned ordinate digit for statistical analysis can be reviewed in Table 1. Adalimumab and certolizumab were the TNF-a inhibitors used. IL-12/23 antagonists utilized included ustekinumab, guselkumab, and risankizumab. IL-17 inhibitors administered counted ixekizumab and secukinumab. These agents were elected independently for each patient based on individual particularities, as well as cost, availability, and preference. The main goal was to provide an overall evaluation of the effectiveness of biologic agents on depression and anxiety; then, secondarily, to ascertain any potential difference between the agents themselves.

The other factors evaluated for an effect on depression and anxiety were patient gender and environment (rural vs. urban). Additionally, we investigated if gender had any influence on psychological status after 6 months of therapy. Table 1 shows the scoring system utilized for statistical analysis.

A Microsoft^®^ Excel for Mac was used to create the data spreadsheet where patients were anonymized. We noted the patient’s gender, environment, PASI and DLQI scores, and depression and anxiety scores. These parameters were given numerical values on an ordinate scale, as seen in Table 1. Furthermore, we noted the patient’s age at the time of referral, as well as the duration (in years) of the disease.

### 2.2. Statistical Analysis

Analysis was performed with the help of SPSS analytical software. To assess the correlation between the presence of psoriasis and the severity of depression and anxiety, we used Pearson Chi-square continuity correction, likelihood ratio, Fisher’s exact test, linear-by-linear association, McNemar’s Test, paired sample *t*-test, related samples Wilcoxon signed-rank test, independent samples Mann–Whitney U test, and independent samples Kruskal–Wallis test.

## 3. Results

### 3.1. Patient and Control Group Demographics 

Within the patient cohort, there were 66 (62.3%) males and 40 (37.7%) females, for a male-to-female ratio of 1.65:1. A total of 36 patients (34%) came from a rural background, whereas 70 (66%) lived in an urban area. Ages at referral varied from 19 to 84 years, with a mean of 49.5 years (SD 14.1 years, 95% CI [46.8–52.2]). Duration of the disease ranged from 1 to 52 years, averaging at 15.3 (SD 10.9 years, 95% CI [13.2–17.3]).

An exact sex, background, and age matching between cases and controls could not be performed. As such, a small imbalance in gender was accepted in order to reach an equal number of controls and a similar age distribution. In the control group, 61 (57.5%) patients were male and 45 (42.5%) were female, for a male-to-female ratio of 1.35:1. A total of 32 (30.2%) and 74 (69.8%) came from a rural and urban background, respectively. Ages ranged from 18 to 74 years, with an average of 43.9 years (SD 14.1 years, 95% CI [41.3–46.6]).

### 3.2. Disease Severity and Symptomology

The lowest PASI score at referral was 13, while the highest reached 33. Meanwhile, DLQI scores ranged from 10 to 30. At 6 months post treatment, these intervals varied from 0 to 8 and from 0 to 9 for PASI and DLQI, respectively.

Thirty-six patients (34%) declared themselves as presenting no symptoms related to depression, 16 (15.1%) as having minimal, 32 (30.2%) mild, 17 (16%) moderate, 4 (3.8%) moderate-to-severe, and 1 (0.9%) having severe depression. Similarly, 48 (45.3%) people with psoriasis reported no anxiety, 36 (34%) mild, 16 (15%) moderate, and 6 (5.7%) reported severe forms of anxiety. In the control group, 72 (67.9%) did not present depression, 18 (17%) minimal, 13 (12.3%) mild, two (1.9%) moderate, and one (0.9%) moderate-to-severe depression. In the same group, 76 (71.7%) did not present anxiety, while 22 (20.8%) and 8 (7.5%) had mild and moderate anxiety, respectively (Table 2).

Regarding treatment, 37 patients (34.9%) received ixekizumab, 21 (19.8%) secukinumab, 5 (4.7%) guselkumab, 5 (4.7%) certolizumab, 7 (6.6%) ustekinumab, 16 (15.1%) risankizumab, and 15 (14.2%) adalimumab. When considering a molecular target, 58 patients (54.7%) were given IL-17 antagonists, 28 (26.4%) received IL-23 antagonists, and the remaining 20 (18.9%), TNF-a inhibitors. Table 3 shows the treatment characteristics within the patent group.

At the 6-month mark, 63 (59.4%) patients were free of depression, with 33 (31.1%), 8 (7.6%), and 2 (1.9%) presenting minimal, mild, and moderate forms, respectively. Concurrently, 81 (76.4%) patients displayed no signs of anxiety, whereas 24 (22.6%) showed mild signs and a single patient (0.9%) complained of moderate anxiety. The results are summarized in Table 4.

### 3.3. Correlation between Psoriasis and Presence of Depression or Anxiety

In the patient group, 66% of patients had depression, in contrast with only 32.1% of controls. Anxiety was present in 54.7% of psoriasis patients, though in only 28.3% of controls. After performing Pearson’s Chi-square test and Fisher’s exact test, we observed a statistically significant correlation between the presence of psoriasis and depression (*p* < 0.0001). The measured odds ratio (OR) was 4.1 (95% CI [2.3–7.3]). Applying the same methods to assess the likelihood of anxiety in these patients, there was a statistically significant correlation between the presence of psoriasis and anxiety (*p* < 0.0001), with an OR of 3.1 (95% CI [1.7–5.4]). In applying McNemar–Bowker Test, it was found that more severe forms of depression and anxiety were significantly more frequent in psoriasis (*p* < 0.05). Using the Chi-square test, we discovered a statistically significant correlation between the presence of depression and anxiety, with a calculated OR of 8.8 (*p* < 0.001).

Despite depressive symptoms being proportionally more frequent in female psoriasis patients (77.5% of patients vs. 59.1% male patients, OR of 2.4), we discovered no significant association between gender and depressive symptoms (*p* = 0.052 > 0.05). Nevertheless, there was a tendency toward significance for *p* < 0.1. Within the control group, depressive symptoms were significantly more frequent in female patients (*p* = 0.019 < 0.05), the OR being 2.7. Alternatively, there was a significant association between anxiety and the female gender in both patient and control groups (*p* = 0.0395 < 0.05 for cases and *p* = 0.006 < 0.01 for controls), the OR being 2.3 and 3.3, respectively.

**Figure 1:**
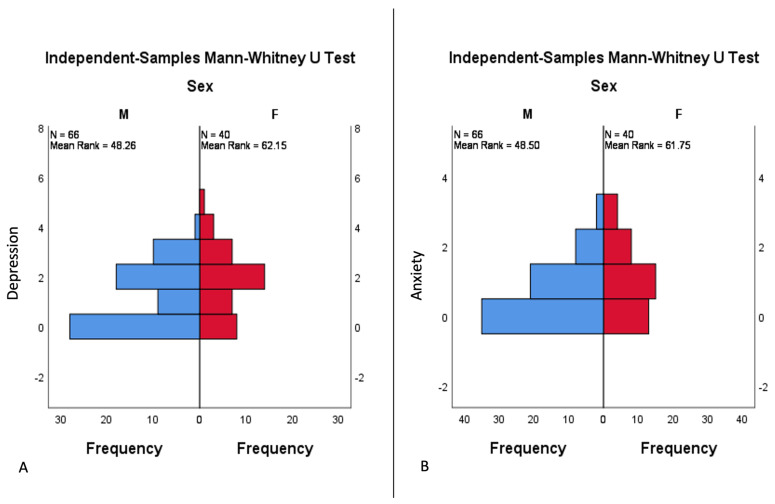
Depression (**A**) and anxiety (**B**) score distribution according to gender in the psoriasis patient group. Based on the independent samples Mann–Whitney U test, there were significantly higher score values in female patients (red horizontal bars) for both depression and anxiety. Abscissa: total frequency of cases: blue bars—male psoriasis patients; red bars—female psoriasis patients. Ordinate: (**A**) depression score from 0 (none) to 6 (severe); (**B**) anxiety score from 0 (absent) to 3 (severe).

After performing the independent samples Man–Whitney U test, we obtained a statistically significant difference in the distribution of depression (*p* = 0.019 < 0.05) and anxiety (*p* = 0.021 < 0.05) scores between the two genders within the patient group. The distribution of depression and anxiety scores shows significantly higher values in females compared to males (Figure 1).

Using the ANOVA test, we obtained a significant association between the duration of psoriasis and the onset of depression (*p* < 0.001). However, employing the same method did not yield any statistically significant associations between disease duration and the presence of anxiety (*p* = 0.8 > 0.05).

### 3.4. Correlation between Disease Severity and Psychologic Morbidity before and after Therapy

**Figure 2:**
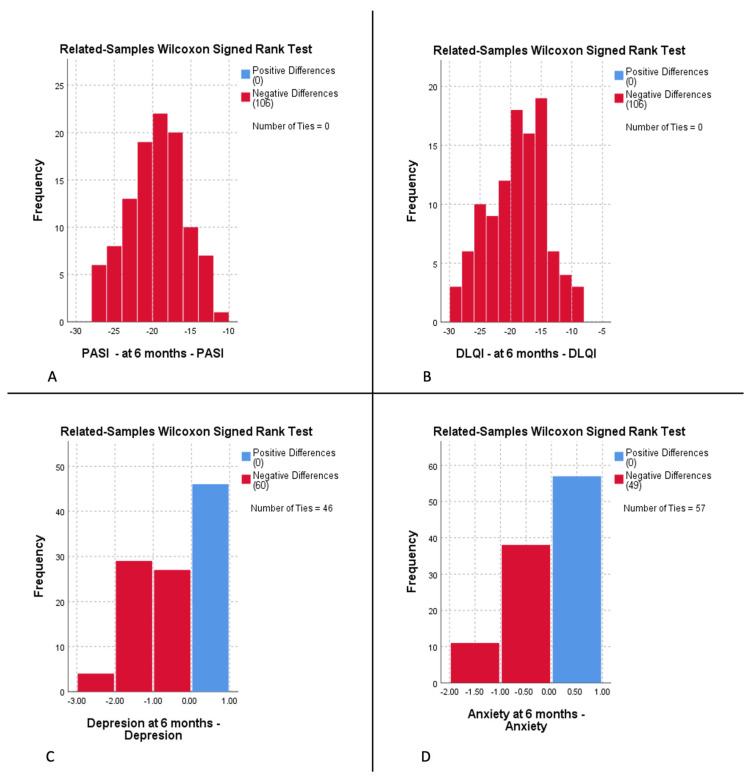
Related samples Wilcoxon signed-rank test histograms for the differences in Psoriasis Area Severity Index (PASI) (**A**), Dermatology Life Quality Index (DLQI) (**B**), depression (**C**), and anxiety (**D**) scores between 6 months and baseline. Vertical blue bars represent the score values that have increased or remained equal from baseline to control (**C**,**D**). Vertical red bars show the score values that decreased in comparison to baseline. All 106 patients presented a decrease in PASI and DLQI scores ((**A**,**B**), respectively). Sixty psoriasis patients had a decrease in depression score, whereas 46 remained equal to baseline (**C**); 49 psoriasis patients presented a diminished anxiety score at study endpoint, and 57 remained unchanged. Abscissa: difference in score. The ordinate displays total number of patients (frequency) of a specific score difference.

Further, by using the ANOVA test, we verified whether there were any correlations between PASI and DLQI scores and the presence and severity of either depression or anxiety. We found a statistically significant link between a higher PASI and DLQI score and the severity of depression (*p* < 0.001), as well as the severity of anxiety (*p* < 0.001). At 6 months of therapy, an improved PASI score resulted in diminished severity of both depression and anxiety (*p* < 0.005). However, an alleviated DLQI score did not result in any statistically significant changes in either depression or anxiety (*p* > 0.955). The related samples Wilcoxon signed-rank test checked for differences between PASI, DLQI, depression, and anxiety scores at baseline and after 6 months of therapy. We rejected the null hypothesis that the median of differences between the two values for each score was 0, with a strong statistical significance (*p* = 0.000) (Figure 2). None of the four parameters presented a score increase compared to the baseline.

Using the paired samples *t*-test for each individual patient at baseline and after 6 months of therapy, we verified for any statistical differences regarding PASI, DLQI, depression, and anxiety scores, respectively. This step pooled all psoriasis patients and did not discriminate according to the specific biologic agent used. For all the aforementioned parameters, we obtained a very strong statistical significance in the difference between baseline and after treatment (*p* < 0.0001). The results of this test are presented in Table 5. 

### 3.5. Efficacy of Biologic Agents in Reducing Psychological Burden

**Figure 3:**
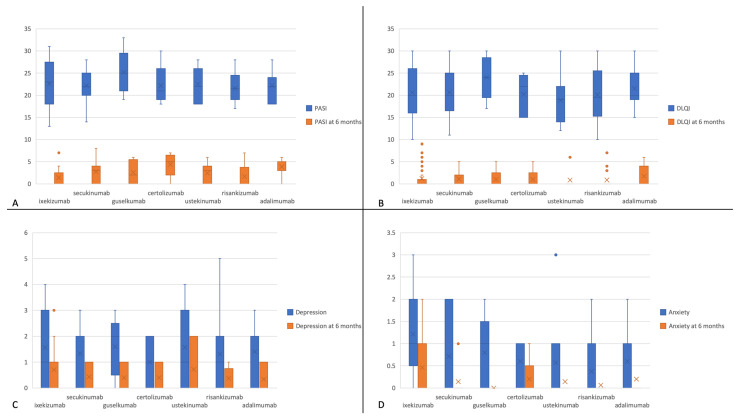
Box plot graphs showing Psoriasis Area and Severity Index (PASI) (**A**), Dermatology Life Quality Index (DLQI) (**B**), depression (**C**), and anxiety (**D**) scores at baseline (blue) and 6-month follow-up (orange) across treatment groups. Abscissa displays the biologic agents used. Values on ordinate represent points on the respective scores.

**Figure 4:**
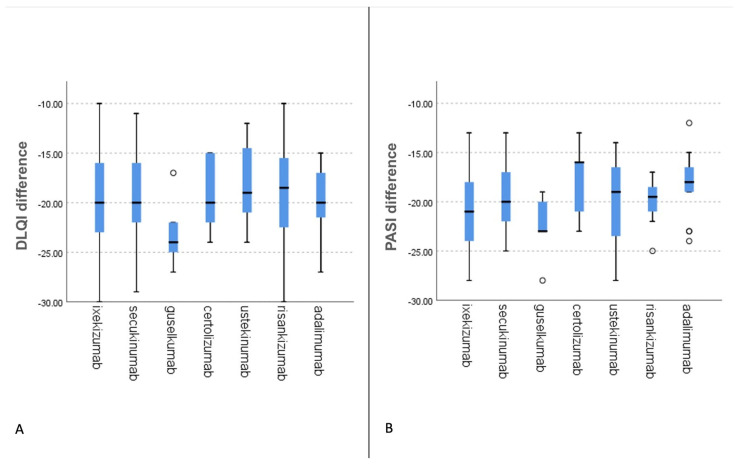
Independent samples Kruskal–Wallis test box plot graphs displaying the reduction of Dermatology Life Quality Index (DLQI) (**A**) and Psoriasis Area and Severity Index (PASI) (**B**) scores across treatment groups. Guselkumab-treated patients displayed the greatest reduction between baseline and 6-month follow-up (non-statistically significant). Abscissa corresponds to the biologic agents used. Ordinate shows the total difference between 6 months and baseline regarding DLQI (**A**) and PASI (**B**) scores, correspondingly.

Last, we tested which of the individual treatment regimens was associated with the best outcome in terms of anxiety and depression scores, as well as PASI and DLQI. This was done by using the independent samples Kruskal–Wallis test. The differences in scores across treatment groups are displayed in Figure 3. Despite there being no statistically significant difference in terms of score reduction across treatment groups (including depression and anxiety scores), guselkumab had the greatest score reduction for both PASI (Figure 4A) (*p* = 0.12 > 0.05) and DLQI (Figure 4B) (*p* = 0.69 > 0.05). However, when taking only the values at 6 months into calculation, ixekizumab was associated with the lowest values in PASI score across treatment groups (*p* = 0.005 < 0.05). Ixekizumab was also correlated with the lowest DLQI score at follow-up, although this was not proven statistically significant (*p* = 0.7 > 0.05).

## 4. Discussion

When comparing the cases and the control groups, there was a statistically significant correlation between the presence of psoriasis and depression and anxiety-related symptoms (OR of 4.1 and 3.1, respectively). This can be interpreted as that, in our series, patients suffering from psoriasis have a 4-fold greater chance of developing depression and 3-fold greater chance of developing anxiety than non-affected individuals. Although rather expectedly, there was a marked association between the presence of depression and anxiety in the cases group, with the OR being 8.8. Additionally, female patients presented with both anxiety and depression more frequently than males, although only the former was statistically significant for *p* < 0.05 (only a weak significance regarding depression at *p* < 0.1). Moreover, the female sex was associated with significantly higher values for depression and anxiety scores within the patient group (both *p* < 0.05). Interestingly, despite these symptoms being less common in the control group, the female sex was still significantly associated with both depression and anxiety when compared to male individuals (OR 2.7 and 3.3, respectively). Duration of disease was correlated with a higher chance of developing depression, although the same could not be said about anxiety.

A higher PASI or DLQI score was also linked to an increased grade of both depression and anxiety (*p* < 0.001), strongly implying that psoriasis severity led to a poorer psychological profile. Our findings also show that there is a clear and strongly statistically significant difference between baseline and post-treatment PASI, DLQI, depression, and anxiety scores for each individual, irrespective of the biologic agent used. This indicates that, across patient groups, biologic therapy for psoriasis is effective in reducing both disease severity and depression and anxiety-related symptoms. Despite not being statistically significant, the fact that Guselkumab was associated with the highest difference may prove a promising starting point for future research on the mechanism of IL-12/23 inhibitors in alleviating psoriasis severity and quality of life.

The association between psoriasis and depression has been intensely studied; however, one between psoriasis and anxiety has not yet been definitively established. Out of their 223 patients, Soliman recounted 47.1% and 32.7% of cases displaying symptoms of depression and anxiety [14]. According to Pollo et al., the prevalence of depression and anxiety in their 281-patient cohort was 19% and 36%, respectively [15]. In the study performed by Bakar et al., 8.5% and 16.9% of individuals in their 174-patient cohort reported depression and anxiety symptoms, respectively [16]. Their findings also strongly correlated with a higher DLQI score, as well as dyslipidemia and lower limb involvement. Even so, there is substantial variation between reported studies with respect to depressive symptoms. Findings generally gravitate towards a 10% prevalence of diagnosed clinical depression and twice as high for depressive symptoms [17]. The widely used Hospital Anxiety and Depression Scale (HADS) questionnaire is a feasible and applicable method of evaluating depressive symptoms, although its use results in the lowest reported percentages of depression and related symptoms. In a study similar to our own, PHQ-9 and GAD-7 were used for 208 Chinese patients, 13.9% being positive for depression and 10.6% for anxiety [18]. In the same study, moderate-to-severe psoriasis was shown to be a positive predictor of moderate-to-severe depression or anxiety symptoms, although longer duration and later start age had a protective function. In a cross-sectional study on 90 patients, the same questionnaires were used to obtain 78.9% and 76.7% prevalence of depression and anxiety, respectively [19]. Furthermore, according to the pilot study by Yu et al., the incidence of anxiety may not be related to the severity of psoriasis when using Zung’s self-rating anxiety scale (SAS) [20].

A recent meta-analysis demonstrated a positive correlation between psoriasis and unspecified anxiety disorder (OR of 1.48 [1.18; 1.85]), as well as between psoriasis and anxiety symptoms (OR of 2.51 [2.02; 3.12]) [21]. However, as the authors remarked, there is an important heterogeneity between studies, explainable in each instance by methodological factors. A similar study performed on 1571 Chinese patients and 1571 controls matched for age and sex showed a higher risk for both depression (adjusted odds ratio (AOR) of 1.85) and anxiety (AOR of 1.47) in individuals with late-onset psoriasis, though with the early-onset form [6]. The reason our results show a higher OR for both comorbidities may be attributable to the heterogeneity of studies populations, as well as the different sample sizes. Another plausible explanation could stem from the differences in cultural influences, with a much higher prevalence of depression and anxiety in the West (circa 4 to 10 times greater) as opposed to Asian countries [22].

Antipsoriatic treatment leads to improvement in PASI, DLQI, as well as depression [23] and anxiety symptoms [24]. According to Tong et al., increased serum levels of TNF-a, IL-17A, and IL-23 were positively linked with HADS depression (HADS-D) scores in psoriasis patients [7]. Concomitantly, psoriasis patients with depression had higher TNF-a, IL-17A, and IL-23 serum levels than those without depression. In the same study, TNF-a, IL-17A, and IL-12 (but not IL-23) serum levels were positively correlated with HADS anxiety (HADS-A) scores, while psoriasis patients with anxiety had increased serum TNF-a and IL-17A compared to those without the psychologic comorbidity. In a similar research study, Tabra et al. discovered increased serum IL-23 in patients with psoriatic arthritis that displayed depression and anxiety-related symptoms [25]. These findings, in addition to the known pathophysiology of psoriasis, may explain the efficacy of biologic therapies in reducing both disease severity and psychological symptoms. Moreover, as Tong and coworkers suggested, TNF-a, IL-17A, and IL-23 could potentially be used as biomarkers for the measurement and monitoring of depression and anxiety in individuals with psoriasis [7].

The positive effect of biologics on reducing depressive symptoms has also been compared to conventional therapies and phototherapy [26]. Within the Psoriasis Longitudinal Assessment and Registry (PSOLAR) study performed by Strober and associates, biologic agents decreased depressive symptoms at a higher rate when compared to standard treatment methods. In a prospective study on Japanese patients with psoriasis, long-term brodalumab therapy managed to significantly decrease the number of patients with anxiety [27]. Interestingly, the same did not apply to patients with depression, even though PHQ-8 scores were significantly reduced at 12 and 48 weeks compared to baseline.

Our research yielded no significant differences in score reduction between biologic agents. As such, our results suggest non-inferiority between treatment regimens. According to a post hoc analysis of the SUPREME study evaluating the efficacy of secukinumab, treatment of psoriasis with this biologic agent for 48 weeks resulted in considerably better skin clearing and a reduction in anxiety and depression symptoms [28]. However, the exacerbation of depression in a patient receiving secukinumab therapy for psoriatic arthritis was also noted [29]. Komori et al. [29] argued that this phenomenon was induced by the drug itself, since secukinumab may transiently increase IL-17 levels after treatment initiation by way of low clearance of the antibody-IL-17 complex. The data from 3 phase 3 RCTs revealed that approximately 40% of patients had remission of depression after 12 weeks of ixekizumab medication, whereas serum levels of C reactive protein improved [30]. Another study showed that guselkumab was more effective in improving depression and anxiety symptoms when compared to placebo or adalimumab [31]. A study comparing risankizumab with ustekinumab or placebo showed a significantly higher proportion of responders to risankizumab in terms of depressive and anxiety symptoms at the 16-week mark [32]. However, differences between the two monoclonal antibodies in this regard became negligible at 52 weeks of treatment. Ustekinumab was proven significantly more effective than placebo in the randomized controlled trial (RCT) conducted by Langley and associates [8]. Similarly, adalimumab was shown to be superior to placebo in the study by Menter et al. [33], whereas Tyring and collaborators found the same for etanercept [34]. 

According to the study by Strober et al., TNF-a inhibitors proved to be particularly effective in reducing depression in psoriasis patients by reducing the serum levels of cytokines that reach the central nervous system [26]. Increased levels of cytokines such as IL-6, IL-10, IL-12, IL-13, and IL-17 were found in individuals with depression [35]. As such, it stands to reason that biologic therapies, which interrupt the inflammatory cycle, may not only alleviate psoriatic lesions but also diminish the psychological burden [36]. TNF-a inhibitors are seemingly the most effective in this regard. However, it should also be noted that, aside from the objective efficacy of biologics, there are certain psychosocial factors associated with psoriasis that can impact anxiety and depression. For example, the visibility of psoriatic lesions may lead to social stigmatization [37,38]. As such, upon reaching a PASI100 (complete disappearance of psoriatic lesions), the quality of life of the patients is also significantly improved. Newer biologics such as IL-17 and Il-23 inhibitors reach PASI100 within the first few months of therapy, also alleviating depression and anxiety-related symptoms [39,40]. Perhaps the reason the patients in our study did not show any significant differences in depression and anxiety score reduction between the seven biologics used is the combined result of inflammation cycle interruption and the psychological effect of visible skin improvement. Therefore, despite their differences in their direct mechanism of action and efficacy in treating the psoriatic lesions themselves, by decreasing PASI and DLQI scores all biologics can be effective in reducing anxiety and depression. Future comparative studies on the efficacies of biologic treatments in psoriasis on alleviating depression and anxiety should be performed.

Despite our research producing no significant correlation between socioeconomic background and the severity of psoriasis, depression, or anxiety, a nationwide population-based study in Israel demonstrated that a poorer socioeconomic status of psoriasis patients led to more severe courses of depression and anxiety [41]. Aside from cultural discrepancies in multi-ethnic patients, a tertiary education level may prove an independent risk factor for depression, while a higher monthly income acts as a protective factor [42]. We believe that access to proper healthcare and education, which is more restricted in rural communities in our country, may also be risk factors. However, a larger sample size is needed to validate this assumption.

IL-17 antagonists such as ixekizumab have been utilized and regulated by the Romanian Ministry of Health starting in 2016 [43], whereas newer agents including IL-23 antagonists were only introduced in 2019 [44]. Due to the COVID-19 pandemic, which started in 2020, the addressability for psoriasis and subsequent prescribing of these agents decreased dramatically. Furthermore, immunosuppressive therapies were only cautiously administered at the beginning of the pandemic until evidence of their safety, particularly when starting biologic agents in bio-naïve patients [45,46,47,48,49]. For this reason, the total number of patients recruited in this study, as well as the ones receiving IL-23 antagonists, is lower than would have been otherwise.

The need to access psychological support for these patients is broad and, in many instances, unmet [1]. Moreover, depressive and anxiety symptoms may be missed by using DLQI alone [50]. These issues are paramount as psoriasis is particularly associated with an increased risk of suicidal ideation [51]. The purpose of this study was to determine if biologic therapies could interrupt the vicious cycle between psoriatic skin lesions and the presence of depression and anxiety. Ascertaining whether the skin disease or the psychological symptoms is often challenging and beyond the scope of this research. Even though the results are promising, a possible limitation of this study may be the relatively low number of patients included. With a greater number of cases, a higher statistical significance in score improvement might have been obtained between specific biologic agents. A recent systematic review of active comparator-controlled clinical trials for psoriasis concluded that the newer biologic agents, namely, IL-17 and Il-12/23 inhibitors, are more effective than TNF-a inhibitors in reducing disease severity [52]. As such, there may be a measurable degree of heterogeneity among the agents used in our research. Additionally, a randomized distribution of the utilized drugs could have provided a more accurate description of these differences. Nevertheless, this study brings clear evidence that, as a whole, biologic agents are effective in reducing both psoriasis severity and improving depression and anxiety-related symptoms. We believe our research may add to the growing body of data supporting biologic therapies as effective in also ameliorating psoriasis-related depressive and anxious symptoms.

## 5. Conclusions

Our prospective study demonstrated that biologic therapy is highly effective in decreasing PASI, DLQI, depression, and anxiety scores at the 6-month follow-up in bio-naïve psoriasis patients. Moreover, our patient series demonstrated that female patients had higher score values significantly more often than male psoriasis patients. Despite no statistically significant difference being found between the agents themselves, Guselkumab was associated with a slightly greater decrease in DLQI and PASI compared to baseline. As such, our results show that all tested biologic agents are seemingly equally efficient in reducing disease severity and psychological symptoms at six months of therapy. 

## Figures and Tables

**Table 1:** Scoring system for statistical Analysis.

Score	Sex	Environment	Depression	Anxiety	Treatment
0	Male	Rural	Absent	Absent	Ixekizumab
1	Female	Urban	Minimal	Mild	Secukinumab
2			Mild	Moderate	Guselkumab
3			Moderate	Severe	Certolizumab
4			Moderate-Severe		Ustekinumab
5			Severe		Risankizumab
6					Adalimumab

**Table 2:** Patient and control demographics—overall (percentages in parenthesis).

		Cases	(%)	Controls	(%)
Sex	Males	66	(62.3)	61	(57.5)
	Females	40	(37.7)	45	(42.5)
Age	Min	19		18	
	Max	84		74	
	Average	49.5		43.9	
Background	Urban	70	(66)	74	(69.8)
	Rural	36	(34)	32	(30.2)
Depression	None	36	(34)	72	(67.9)
	Minimal	16	(15.1)	18	(17)
	Mild	32	(30.2)	13	(12.3)
	Moderate	17	(16)	2	(1.9)
	Moderate-to-severe	4	(3.8)	1	(0.9)
	Severe	1	(0.9)	0	0
Anxiety	None	48	(45.3)	76	(71.7)
	Mild	36	(34)	22	(20.8)
	Moderate	16	(15)	8	(7.5)
	Severe	6	(5.7)	0	0

**Table 3:** Treatment characteristics in patient group (percentages in parenthesis).

		No.	(%)
Treatment	Ixekizumab	37	(34.91)
	Secukinumab	21	(19.81)
	Guselkumab	5	(4.72)
	Certolizumab	5	(4.72)
	Ustekinumab	7	(6.6)
	Risankizumab	16	(15.09)
	Adalimumab	15	(14.15)
Biologic agent class	IL-17 antagonist	58	(54.7)
	IL-23 antagonist	28	(26.4)
	TNF-a inhibitor	20	(18.9)
Depression (post treatment)	None	63	(59.43)
	Minimal	33	(31.13)
	Mild	8	(7.55)
	Moderate	2	(1.89)
	Moderate-to-severe	0	0
	Severe	0	0
Anxiety (post treatment)	None	81	(76.42)
	Mild	24	(22.64)
	Moderate	1	(0.94)
	Severe	0	0

**Table 4:** Detailed characteristics of patient group.

	Age	PASI	DLQI	Depression	Anxiety	Depression at 6 mo.	Anxiety at 6 mo.	PASI at 6 mo.	DLQI at 6 mo.
Mean	49.5	22.4	20.7	1.4	0.8	0.5	0.3	2.3	1.2
Std. Error of Mean	1.4	0.4	0.5	0.1	0.1	0.1	0	0.2	0.2
Median	49	22	20	2	1	0	0	2	0
Mode	41	18	20	0	0	0	0	0	0
Std. Deviation	14.1	4	5.6	1.2	0.9	0.7	0.5	2.4	2.1
Variance	199.9	15.9	30.9	1.6	0.8	0.5	0.2	5.7	4.5
Skewness	0.1	0.2	0.1	0.4	0.9	1.3	1.5	0.5	1.7
Std. Error of Skewness	0.2	0.2	0.2	0.2	0.2	0.2	0.2	0.2	0.2
Kurtosis	−0.7	−0.5	−0.9	−0.8	−0.1	1.4	1.1	−1.1	
Std. Error of Kurtosis	0.5	0.5	0.5	0.5	0.5	0.5	0.5	0.5	0.5
Range	65	20	20	5	3	3	2	8	9
Minimum	19	13	10	0	0	0	0	0	0
Maximum	84	33	30	5	3	3	2	8	9

Abbreviations: PASI—Psoriasis Area and Severity Index; DLQI—Dermatology Quality of Life Index; 6 mo.—6 months; std.—standard.

**Table 5:** Paired samples *t*-test for PASI, DLQI, depression, and anxiety score difference at baseline and 6 months of therapy.

	Mean	SD	Std. Error Mean	95% CI of the Difference		*t*	Sig. (2-Tailed)
				Upper	Lower		
PASI difference	20.1	3.8	0.4	19.3	20.8	54.2	*p* < 0.0001
DLQI difference	19.5	4.8	0.5	18.6	20.4	42.3	*p* < 0.0001
Depression score difference	0.9	0.9	0.1	0.7	1.1	10.2	*p* < 0.0001
Anxiety score difference	0.6	0.7	0.1	0.4	0.7	8.6	*p* < 0.0001

Abbreviations: PASI—Psoriasis Area Severity Score; DLQI—Dermatological Life Quality Index; 6 mo.—6 months of therapy; SD—standard deviation; CI—confidence interval; *t—t*-test.

## Data Availability

The data contained in the study can be obtained from the corresponding author upon reasonable request.
